# Assessment of Long-Term Radiological Effects on Plants and Animals from a Deep Geological Repository: No Discernible Impact Detected

**DOI:** 10.1007/s13280-013-0403-9

**Published:** 2013-04-26

**Authors:** Jesper Torudd, Peter Saetre

**Affiliations:** 1 Facilia AB, Gustavslundsvägen 151C, 167 51 Stockholm, Sweden; 2 Swedish Nuclear Fuel and Waste Management Co. (SKB), Box 250, 101 24 Stockholm, Sweden

**Keywords:** ERICA Tool, Reference organism, Keystone species, ICRP, Reproduction, Mortality

## Abstract

This study investigates whether non-human biota are protected against harmful effects of ionizing radiation after a possible future release of radioactive matter from a planned repository for spent nuclear fuel. Radiation dose rates to a broad spectrum of organisms were calculated based on data from sampled organisms and modeled activity concentrations. Calculations were performed with the ERICA Tool, a software program which applies a screening dose-rate value of 10 microgray per hour (μGy h^−1^) for all types of organisms. Dose rates below this value are thought to result in minimal risk to the individual or population. All calculated dose rates were below the screening value and below the lowest relevant band of “derived consideration levels” proposed by the International Commission on Radiological Protection. This provides a sound basis for arguing that no individuals or populations of examined species would be harmfully affected by a possible radioactive release from the repository.

**Electronic supplementary material:**

The online version of this article (doi:10.1007/s13280-013-0403-9) contains supplementary material, which is available to authorized users.

## Introduction

Attitudes concerning the protection of animals and plants from deleterious effects of ionizing radiations have changed considerably over the last 35 years. Up until around 1975, the issue was entirely ignored. As a next stage, the 1977 Recommendations of the International Commission on Radiological Protection (ICRP 1977) made the assumption that if man is adequately protected, then other living things are also likely to be sufficiently protected, and essentially the same attitude was taken in the 1990 ICRP Recommendations (ICRP 1991). The 2007 ICRP Recommendations (ICRP 2007), however, include an acknowledgment of the need for a systematic approach for radiological assessment of non-human species. This was not driven by any particular concern over environmental radiation hazards. It was meant to fill a conceptual gap in radiological protection, and to develop a protection policy in line with society’s general goals for environmental protection (ICRP 2003).

However, the objectives of such a protection policy for non-human biota are not yet as clear as those of human radiological protection, which aims to prevent deterministic tissue reactions and reduce the risk of stochastic effects to as low as reasonably achievable. ICRP (2007) suggests that the aim should be a negligible effect on the maintenance of biological diversity, the conservation of species, and the health and status of natural habitats, communities, and ecosystems—i.e., on the population level. According to ICRP (2008), detectable effects in some members of a population would not necessarily lead to a consequence for the population. Instead, the biological endpoints of most relevance in individuals after radiation exposure will be those that could lead to changes in population size or structure.

ICRP (2008) goes on to say that some form of practical means is required to translate knowledge of the effects of radiation on different types of animals and plants into advice on management decisions and judgments that may be needed. To this end, ICRP proposes the use of a limited set of reference animals and plants to serve as a basis for the understanding and interpretation of the relationships between exposure and dose, and between dose and certain categories of effect, for a few, clearly defined types of animals and plants.

Furthermore, ICRP (2008) notes that “dose limits” of the form used in human radiological protection would be inappropriate, but that some form of numerical guidance is required. However, no single dose-rate value is relevant, due to the variation in amount and type of information available and the differences in radiation sensitivity between species. Instead, ICRP (2008) sets out proposed bands of “derived consideration reference levels.” Within these bands, it is likely that there is some probability of the induction of deleterious effects of ionizing radiation in the pertinent reference animals or plants, and this probability could be taken into account in the optimization of protection (by inference, below these bands the risks would appear to be negligible and do not need to be taken into account in optimization).

In parallel and aligned with these developments, a series of major research projects (EPIC, FASSET, ERICA, PROTECT) concerning these issues has been funded under the European Commission EURATOM Framework programs. An overview of the entire series and detailed descriptions of each project, including links to the resulting scientific publications, are available at the ERICA web site (see erica-project.org). The project program generated the ERICA Integrated Assessment approach and the ERICA Tool used in this study as described in Box S1 (in Electronic Supplementary Material) and in more detail in Brown et al. (2008).

The ERICA Tool uses a default screening dose rate of 10 microgray per hour (μGy h^−1^) to assist in the separation of situations of negligible concern from those situations where it is appropriate to pause for reflection to consider whether any concern is warranted. The ERICA default screening dose rate is, generally speaking, at the lower end of the bands of derived consideration reference levels defined by ICRP (2008), and can provide input to the optimization of radiological protection of the environment in planned exposure situations.

This dose rate was originally derived as a predicted no-effect-dose-rate value for ecosystems, based on a distribution analysis of mortality and reproduction response to chronic exposure in a broad range of organisms (Garnier-Laplace and Gilbin 2006). In subsequent analyses restricted to vertebrates, invertebrates, and plants, the use of this screening dose rate was further supported, and it can be interpreted as the dose rate where 5% of species are expected to have a 10% reduction in reproductive rate, accounting for data uncertainties (Andersson et al. 2009; Garnier-Laplace et al. 2010).

The current awareness of environmental protection issues has emerged over about a decade, and the policy advice of ICRP and the practical tools provided by the EURATOM research framework programs were generated over that time-scale. Recently, the ERICA Tool has been used to assess the radiological impact and risk to non-human biota associated with an existing repository for low-level radioactive waste and with routine liquid discharges of nuclear power plants (LLWR 2011; Vandenhove et al. 2012). Robinson et al. (2010) describe the use of the ERICA Tool for an analysis of the impact on non-human biota of a “generic” geological disposal facility.

The Swedish Nuclear Fuel and Waste Management Company (SKB) has submitted applications to build a final repository for spent nuclear fuel at Forsmark, Sweden. To comply with regulatory requirements (SSM 2008), this study of the possible effects on non-human biota of future releases of radioactive material from the planned repository was performed as part of the safety assessment provided by SKB (2011). To the best of the authors’ knowledge, this is the first case where the policy of ICRP and the ERICA Tool has been applied in a formal license application concerning a repository for spent nuclear fuel.

## Materials and Methods

The effects on the environment were assessed by evaluating the potential effects of a radionuclide release on individual organisms. The rationale for this approach is the assumption that if there are no detrimental effects at the level of individuals, then negative consequences at the population, community, or ecosystem levels can also be excluded.

### Assessment Methodology

**Fig. 1:**
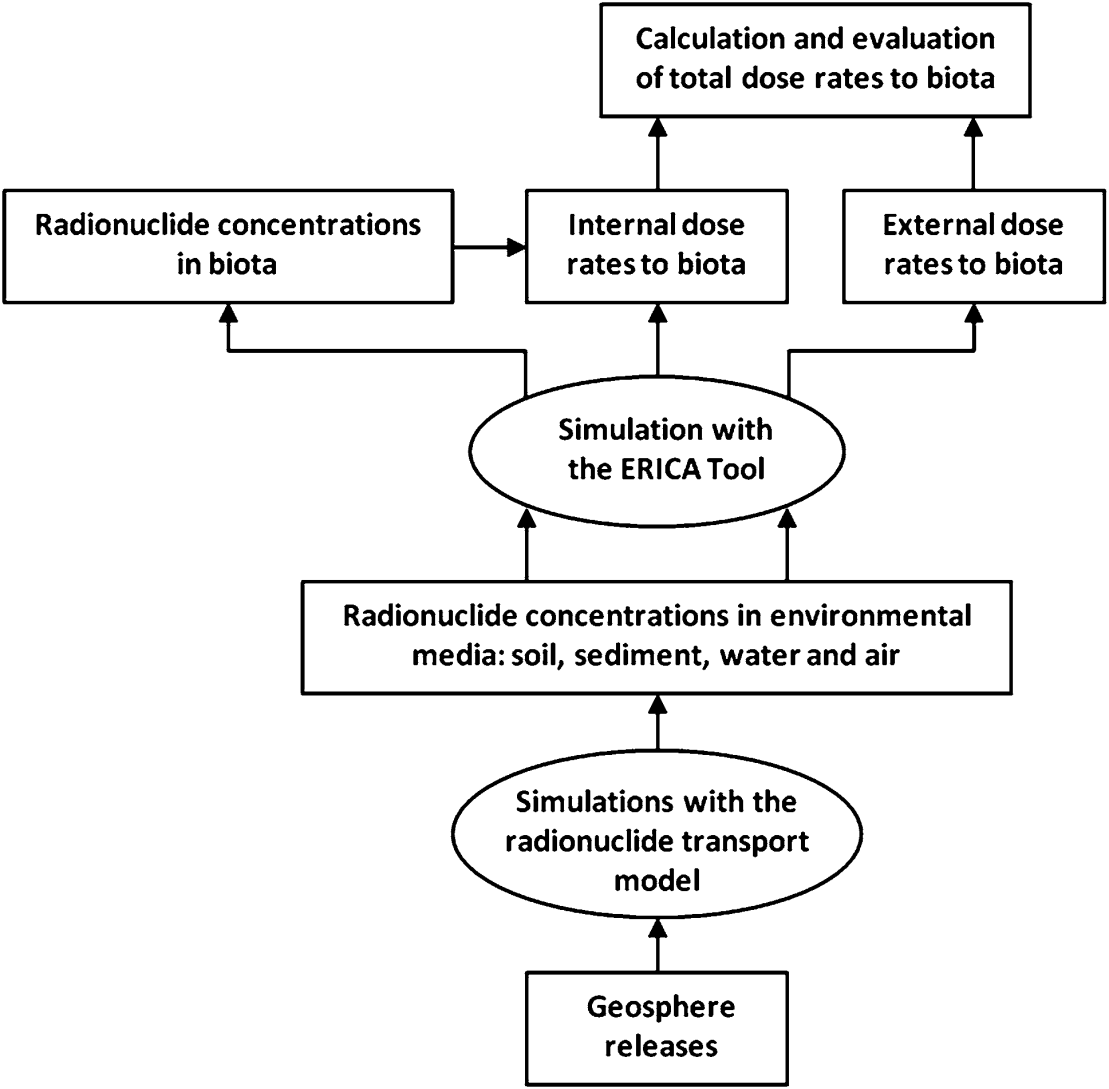
Assessment of the consequences of radionuclide releases for non-human biota. The assessment starts by calculating radionuclide concentrations in environmental media. These concentrations are then used as input data to the ERICA Tool, and the resulting dose rates are compared with a screening dose rate. Each step in the calculation procedure are explained in SKB (2010, Sects. 11.2.1–11.2.8), and details are provided in Torudd (2010)

Figure 1 summarizes the logic of this study. The basic assumption of the SKB safety assessment is that some degree of failure of the barriers at the repository will lead to a release of radionuclides (SKB 2011). The scenario that results in the largest predicted release of radionuclides to the biosphere is canister failure as a result of enhanced corrosion. This would be due to advective conditions in the deposition hole following the loss of buffer through erosion. The outcome of such an event is illustrated by the release from central corrosion case (SKB 2011). The activity concentrations in the environment that would result from such a release constitute the primary input for the present assessment.

To calculate activity concentrations in required environmental media, we considered the long-term effects of radionuclide-bearing groundwater discharge to the future Forsmark landscape. The maximum release of each radionuclide from the far-field geosphere during a period of 1 million years (SSM 2008; SKB 2011) was used as a constant-rate source term to the biosphere during a full interglacial episode (9000 bc to 9400 ad), and the transfer and accumulation of radionuclides in ecosystems were simulated for several potential discharge areas (Berglund et al. 2013). As shoreline displacement and accumulation/erosion processes are expected to drive the development of a discharge area, from a sea basin, through a lake-mire complex, to a mire (Lindborg et al. 2013), the activity concentrations in the environment of each discharge area were simulated dynamically as a function of a continuous landscape succession (Avila et al. 2013).

A potential release of radionuclides could also occur when the site is covered by ice. However, terrestrial biota are expected to be scarce during a glacial episode, and radionuclide concentrations in the sea at the ice margin are expected to be lower than during an interglacial episode (due to the high water exchange associated with open sea basins). Thus, radionuclide concentrations from an interglacial episode were considered sufficient for the assessment of radiological protection of the environment.

For each radionuclide the maximum concentrations in the upper regolith (soil and sediment), air (for C-14), and water (freshwater and marine/brackish) over all potential discharge areas during the simulation period were used as the required input values in the assessment (Table S1 in Electronic Supplementary Material). We then used the ERICA Tool to calculate the internal dose rates to biota from modeled activity concentrations in the organisms, and to estimate the external dose rates from the activity concentrations in the environment. Dose rates were weighted as appropriate using the ERICA default radiation weighting factors of 10 for alpha, 3 for low energy beta, and 1 for other beta and gammas. The numerical endpoint of the consequence assessment was the total absorbed dose rate to each selected organism from each radionuclide considered in the assessment. Finally, the sum of the dose rates over all radionuclides was evaluated against a screening dose rate of 10 μGy h^−1^, which corresponds to the predicted no-effect dose rate, below which essentially no effects on individual organisms or populations are expected (cf. Garnier-Laplace et al. 2010).

### Radionuclides Considered in the Assessment

All radionuclides that reach the biosphere from the far-field geosphere under the central corrosion case (SKB 2011) were considered in the assessment. Four radionuclides (Ac-227, Pa-231, Pd-107, and Sn-126) were excluded from the analysis as neither site nor literature data were available with respect to biological uptake (i.e., concentration ratios, CR). The assessment included 27 radionuclides (of 18 elements), all present in the ERICA database, either by default or (shown in italics in Table S1 in Electronic Supplementary Material) added from the ERICA optional set.

### Organisms Considered in the Assessment

**Fig. 2:**
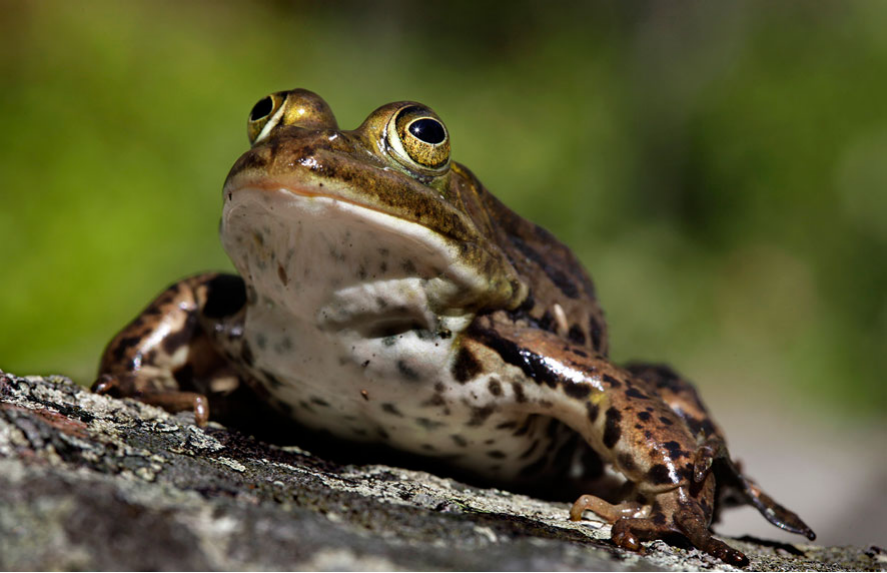
Pool frog (*Rana lessonae*) is an endangered species presently found in shallow wetland pools in the Forsmark area. Corresponding reference organism (Table 2): Amphibian. Photographer: Lasse Modin. Reproduced with permission from SKB picture bank

To prevent or reduce the frequency of deleterious radiation effects in the environment to a level where they would have a negligible impact on the maintenance of biological diversity, the conservation of species, or the health and status of natural habitats, communities and ecosystems, it is necessary to relate exposure to dose, dose to effect, and effect to consequences (ICRP 2008). To permit such analyses, the ERICA Tool uses a small, well-defined set of *reference organisms*. Each reference organism has its own specified geometry and habitat in terrestrial, freshwater, or marine ecosystems (cf. Fig. 2). The approach is compatible with that used by ICRP, and some of the geometries proposed for the “reference animals and plants” of ICRP (2008) are used as defaults in the ERICA Tool.

According to Beresford et al. (2007), the selection of reference organisms included in the ERICA Tool makes it possible to address all protected species within Europe. Nevertheless, to increase the confidence in the analysis, a number of common species currently found in Forsmark were also included. Species that are presently found in the area were the primary target of the SKB safety assessment.

Several additional organisms were considered to satisfy licensing conditions set by the regulatory authority (SSM 2008). Thus, to ensure that ecosystem functioning is protected, organisms playing a critical role (keystone species) or being very abundant (foundation species) and representing different functional groups were identified. Furthermore, species that have an economic importance to man (e.g., fish and game) or a conservation value (e.g., endemic and endangered species) were identified. These organisms were considered for conceptual illustration purposes rather than in view of each particular species as such.

The assessment was limited to species occurring in marine/brackish and freshwater ecosystems and in wetlands, as it was expected that these are the natural ecosystem types that may be most affected by discharge of contaminated groundwater, given the expected landscape evolution over the considered timescales (Lindborg 2010). Agricultural ecosystems were not considered in the analysis. This was because future-contaminated agricultural land in Forsmark is likely to originate from drained mires, and these agricultural soils are expected to be productive (and thus provide a stable environment) for 100 years or less (Lindborg 2010, Sects. 5.5 and 5.7). Thus, the species associated with this land would either be introduced by humans (crop or livestock) or invade from adjacent agricultural land, and consequently they would be part of large and stable populations which would be marginally affected by potential radiological effects in an individual (and transient) habitat patch.

A considerable body of data concerning plants and animals collected in the Forsmark area was available (for details, see Torudd 2010). However, these samples were collected for purposes other than this study, and neither site nor literature data were available for most of the identified species of interest. Instead, each such species was mapped to a reference organism of similar taxonomy within the appropriate ecosystem. This mapping of keystone and foundation species and economically important species can be found in Table 1. Identified endangered species (~100 species) and their corresponding reference organisms are listed in Torudd (2010).

**Table 1:** Organisms (from three ecosystems—terrestrial, freshwater, and marine/brackish) with important ecosystem functions and/or of economic value in the Forsmark area. English and scientific names are listed together with the corresponding ERICA Reference Organisms. Modified from Torudd (2010)

English names	Scientific names	ERICA reference organisms
Terrestrial		
Alder	*Alnus glutinosa*	Tree
Bottle sedge	*Carex rostrata*	Grasses and herbs
Cloudberry^a^	*Rubus chamaemorus*	Grasses and herbs
Common frog	*Rana temporaria*	Amphibia
Cranberry^a^	*Vaccinium oxycoccus*	Grasses and herbs
Norwegian spruce^b^	*Picea abies*	Tree
Peat moss^b^	*Sphagnum* sp.	Lichens and bryophytes
Pine tree^b^	*Pinus sylvestris*	Tree
Red fox	*Vulpes vulpes*	Mammal
Reed	*Phragmites australis*	Grasses and herbs
Sedges^a^	*Carex* sp.	Grasses and herbs
Water vole	*Arvicola terrestris*	Mammal
Freshwater		
Microphytobentos	–	Phytoplankton
Midge	*Tanypodinae*	Insect larvae
Perch^b^	*Perca fluviatilis*	Pelagic fish
Pike^b^	*Esox lucius*	Pelagic fish
Reed	*Phragmites australis*	Vascular plant
Roach	*Rutilus rutilus*	Pelagic fish
Ruffe	*Gymnocephalus cernua*	Pelagic fish
Stoneworts	*Chara* sp.	Vascular plant
Tench^b^	*Tinca tinca*	Pelagic fish
Marine/brackish		
Baltic macoma	*Macoma balthica*	Benthic mollusc
Bladder wrack	*Fucus vesiculosus*	Macroalgae
Burbot^a^	*Lota lota*	Benthic fish
Common eider^a^	*Somateria mollissima*	Bird (duck)
Duck mussel	*Anodonta anatina*	Bivalve mollusc
Eel^a^	*Anguilla anguilla*	Pelagic fish
Herring^b^	*Clupea harengus*	Pelagic fish
Idothea	*Idothea* sp.	Crustacean
Lumpsucker^a^	*Cyclopterus lumpus*	Benthic fish
Perch^b^	*Perca fluviatilis*	Pelagic fish
Phytoplankton	–	Phytoplankton
Pike^b^	*Esox lucius*	Pelagic fish
Ringed seal^a^	*Pusa hispida*	Mammal
Tench^b^	*Tinca tinca*	Pelagic fish
Zooplankton	–	Zooplankton

^a^Species with an economic value but not deemed critical for ecosystem function

^b^Species important for ecosystem function and having an economic value

For most of the species sampled at the site, CRs for a number of radionuclides (see below) and morphology were available from the site investigation or could be determined. However, most species were represented by a few individuals only (typically three or less). Thus, it was unlikely that the field data would capture the large inherent variability in the equilibrium CRs at the species level (Sheppard 2005), and consequently the assessment was primarily founded on ERICA reference organisms and the default CR values associated with them. However, so as not to overlook site-specific characteristics of bioaccumulation, we also examined dose rates for ERICA reference organisms calculated with CRs from site data aggregated to the appropriate level (as far as possible).

### Representation of Organisms

There is an enormous diversity of organisms with respect to size, morphology, and habitat choice. The representative organisms used in ERICA and the reference animals and plants used by ICRP are carefully selected to represent different organism sizes, different trophic levels, different ecosystem positions, etc. The choice of organisms is amply described in Brown et al. (2008) and in ICRP (2008).

### Activity Concentrations in Biota

Plant root uptake from contaminated soil, ingestion of contaminated food and water, and inhalation of contaminated air will result in an internal activity concentration of radionuclides. In the ERICA Tool, whole-body activity concentrations in biota are predicted directly from the activity concentrations in the environmental media, using equilibrium CRs. For terrestrial biota, the CRs are defined as the radionuclide activity concentration in whole body (Bq kg^−1^ fresh weight) divided by the radionuclide activity concentration in soil (Bq kg^−1^ dry weight) or in air (Bq m^−3^). For aquatic biota, the CRs are defined as the activity concentration in whole body (Bq kg^−1^ fresh weight) divided by the activity concentration in filtered water (Bq l^−1^).

Most CRs estimated in this study are based on measured values of stable element concentrations in biota and environmental media (Tröjbom and Nordén 2010; Tröjbom and Grolander 2010).

### Dosimetry

Radionuclides in the environment lead to both internal and external exposure of organisms. In the ERICA Tool, the *internal* absorbed dose rate (μGy h^−1^) in biota is a function of whole-body activity concentration (see above), size of the organism and the types, yields, and energies of emitted radiations. Absorbed dose rate from *external* radiation depends not only on organism size and the types, yields, and energies of emissions but also on the contamination level in and the properties of the environment, but is not dependent on the activity concentration in the organism. Below is a brief description of the methods used for calculating dose conversion factors in the ERICA Tool. A detailed description of the underlying approaches and the data that have been applied in the dosimetric module of the ERICA Tool is presented in Ulanovsky et al. (2008).

#### Calculation of Dose Conversion Coefficients (DCC)

Dose coefficients are quantities linking amounts or concentrations of activity to doses or dose rates. In the ERICA Tool, two sets of DCCs are defined: for doses due to intakes, DCC_int_ is defined as the internal absorbed dose rate (μGy h^−1^) per unit activity concentration in an organism (Bq kg^−1^ fw) and for doses due to exposures from surrounding media, DCC_ext_ is defined as the external absorbed dose rate (μGy h^−1^) per unit concentration in environmental media (Bq kg^−1^ or Bq l^−1^ fw) (Pröhl 2003; Brown et al. 2008). Using DCC_int_ and DCC_ext_, internal and external dose rates to an organism can be computed; the total dose rate to an organism is obtained as the sum of these dose rates.

## Results

ERICA Tier 2 assessments generate risk quotients (RQs, i.e., estimated dose rate/“screening” dose rate) and ERICA Tier 3 assessments provide dose rates (cf. Box S1 in Electronic Supplementary Material). Table 2 lists these data, obtained for reference organisms in the Forsmark area. For all investigated organisms, the “expected” RQs were 10^−4^ or smaller and calculated dose rates were at least four orders of magnitude below the screening dose level of 10 μGy h^−1^ (Table 2).

**Table 2:** Risk quotients (RQ) and whole-body dose rates (μGy h^−1^) for terrestrial, freshwater, and marine/brackish reference organisms in the Forsmark area, given the release assumed in the safety assessment. RQs are estimated dose rates divided by the “screening dose rate,” “expected,” and “conservative” RQs are explained in Box S1 (in Electronic Supplementary Material). Estimated dose rates from deterministic calculations are given together with the 95th percentile from probabilistic simulations. Modified from Torudd (2010)

Reference organisms	Tier 2 RQ values		Tier 3 dose rates (μGy h^−1^)	
	Expected	Conservative	Deterministic estimates	95th percentile
Terrestrial				
Amphibia	2.9 × 10^−6^	8.7 × 10^−6^	2.9 × 10^−5^	5.9 × 10^−5^
Bird	2.7 × 10^−6^	8.1 × 10^−6^	2.7 × 10^−5^	5.9 × 10^−5^
Detritivorous invertebrate	6.4 × 10^−6^	1.9 × 10^−5^	6.4 × 10^−5^	1.4 × 10^−4^
Flying insect	6.1 × 10^−6^	1.8 × 10^−5^	6.1 × 10^−5^	1.4 × 10^−4^
Gastropod	5.9 × 10^−6^	1.8 × 10^−5^	6.0 × 10^−5^	1.4 × 10^−4^
Grasses and herbs	3.7 × 10^−6^	1.1 × 10^−5^	3.6 × 10^−5^	8.5 × 10^−5^
Mammal, large	2.2 × 10^−6^	6.5 × 10^−6^	2.2 × 10^−5^	4.9 × 10^−5^
Mammal, small	2.5 × 10^−6^	7.6 × 10^−6^	2.5 × 10^−5^	5.2 × 10^−5^
Lichen and bryophytes	6.7 × 10^−5^	2.0 × 10^−4^	6.7 × 10^−4^	1.2 × 10^−3^
Reptile	2.9 × 10^−6^	8.6 × 10^−6^	2.8 × 10^−5^	5.8 × 10^−5^
Shrub	7.6 × 10^−6^	2.3 × 10^−5^	7.4 × 10^−5^	2.0 × 10^−4^
Soil invertebrate	6.3 × 10^−6^	1.9 × 10^−5^	6.3 × 10^−5^	1.4 × 10^−4^
Tree	6.1 × 10^−6^	1.8 × 10^−5^	5.9 × 10^−5^	1.6 × 10^−4^
Freshwater				
Bird	1.6 × 10^−6^	4.9 × 10^−6^	1.6 × 10^−5^	4.4 × 10^−5^
Bivalve mollusc	3.7 × 10^−5^	1.1 × 10^−4^	3.7 × 10^−4^	7.7 × 10^−4^
Crustacean	2.7 × 10^−5^	8.1 × 10^−5^	2.7 × 10^−4^	4.8 × 10^−4^
Gastropod	2.7 × 10^−5^	8.2 × 10^−5^	2.7 × 10^−4^	5.3 × 10^−4^
Insect larvae	1.9 × 10^−4^	5.6 × 10^−4^	1.9 × 10^−3^	5.1 × 10^−3^
Mammal	1.8 × 10^−6^	5.4 × 10^−6^	1.8 × 10^−5^	4.3 × 10^−5^
Pelagic fish	1.7 × 10^−6^	5.0 × 10^−6^	1.7 × 10^−5^	4.0 × 10^−5^
Phytoplankton	3.4 × 10^−4^	1.0 × 10^−3^	3.4 × 10^−3^	9.7 × 10^−3^
Vascular plant	5.2 × 10^−5^	1.6 × 10^−4^	5.2 × 10^−4^	1.2 × 10^−3^
Zooplankton	2.2 × 10^−5^	6.5 × 10^−5^	2.2 × 10^−4^	4.9 × 10^−4^
Marine/brackish				
Benthic fish	1.1 × 10^−7^	3.4 × 10^−7^	1.1 × 10^−6^	1.8 × 10^−6^
Benthic mollusc	1.9 × 10^−7^	5.6 × 10^−7^	1.9 × 10^−6^	3.1 × 10^−6^
Bird	3.2 × 10^−8^	9.5 × 10^−8^	3.2 × 10^−7^	9.0 × 10^−7^
Crustacean	6.3 × 10^−8^	1.9 × 10^−7^	6.3 × 10^−7^	1.2 × 10^−6^
Macroalgae	1.4 × 10^−7^	4.3 × 10^−7^	1.4 × 10^−6^	1.8 × 10^−6^
Mammal	8.5 × 10^−9^	2.5 × 10^−8^	8.5 × 10^−8^	2.2 × 10^−7^
Pelagic fish	3.4 × 10^−8^	1.0 × 10^−7^	3.4 × 10^−7^	1.0 × 10^−6^
Phytoplankton	2.9 × 10^−7^	8.7 × 10^−7^	2.9 × 10^−6^	6.2 × 10^−6^
Polychaete worm	3.0 × 10^−7^	8.9 × 10^−7^	3.0 × 10^−6^	4.6 × 10^−6^
Vascular plant	1.3 × 10^−7^	4.0 × 10^−7^	1.3 × 10^−6^	1.9 × 10^−6^
Zooplankton	3.4 × 10^−8^	1.0 × 10^−7^	3.4 × 10^−7^	6.9 × 10^−7^

According to the general criteria proposed for the ERICA Tool, it would thus have been sufficient to terminate the analysis at Tier 2. However, as there are rather few earlier studies of a similar nature, we wanted to investigate further the potential distribution in dose rates. Also, by performing Tier 3 calculations the effects of uncertainties in the assumptions and input data are, to some extent, assessed.

When the uncertainty of the dose rates was taken into account (using the “conservative” RQ or by using the 95th percentile of the probabilistic dose-rate simulations), the dose rates were still far below the screening dose rate. Thus, for freshwater phytoplankton as the organism that received the highest dose rate, the deterministic dose rate was 3 × 10^−3^ μGy h^−1^, corresponding to a RQ of 3 × 10^−4^. The “conservative” risk quotient was 1 × 10^−3^ and the 95th percentile of the dose rate was below 0.01 μGy h^−1^.

The mean values from the probabilistic calculations were almost identical to the deterministic estimates (data not shown), which was expected as the arithmetic mean values from the probability density functions of the CR parameters were used in the deterministic calculations.

No substantive difference in calculated dose rates could be detected for ERICA reference organisms when CRs from the site were used (as far as possible) compared with calculations based entirely on generic data. Thus, the use of site data did not appreciably affect the calculated dose rates. This may, however, partly reflect that site data were not complete with respect to dose-contributing radionuclides.

Comparisons of the CRs from site data with CR for comparable ERICA reference organisms showed that CRs from the site were typically captured within the reported values for the corresponding reference organism for well-investigated organisms (e.g., terrestrial vascular plants and marine pelagic fish) and elements. However, for organism groups that were less well represented in the database, CRs for individual species from the site frequently fell outside the 95 % interval of the distribution for the corresponding ERICA reference organism. This pattern was seen for all three ecosystems. In most cases, systematic differences could be attributed to the limited sample size or lack of representative samples in the ERICA database, but in a few cases there were indications that CRs at the site showed site-specific characteristics (Torudd 2010; Tröjbom and Grolander 2010, Tröjbom and Nordén 2010). For example, the CRs for Cs and Pb in terrestrial plants and mammals from the site tended to be systematically lower than in the ERICA database, and a similar trend was seen for the CRs for U in freshwater fish and plants (Table 3). On the other hand, the CR values for marine mollusks at the site were systematically higher than the corresponding CRs for reference organisms for several elements, though these differences were typically within an order of magnitude.

**Table 3:** Comparison of concentration ratios (CR) observed in species sampled from Forsmark and CRs supplied in the ERICA Tool for reference organisms. CR for terrestrial organisms (A) are computed as kg dw/kg fw and are, thus, dimensionless; the unit of CRs for aquatic organisms (B) is l/kg fw

A								
Element	Terrestrial mammals from site			Reference mammal^a^	Terrestrial plant from site			Reference grass or herb
	Common shrew	Moose	Yellow-necked mouse		Bilberry	Small cow-wheat	Stone bramble	
Cl	5.0E+01	4.4E+00	2.5E+01	7.0E+00	5.3E+01	7.2E+01	1.9E+02	1.7E+01
Cs	3.7E−02	3.6E−02	9.2E−02	2.9E+00	1.5E−01	2.2E−01	7.7E−03	6.9E−01
I	1.4E−01	–	–	4.0E−01	4.2E−02	3.8E−02	2.2E+00	1.4E−01
Nb	6.0E−04	4.1E−05	–	1.9E−01	4.5E−04	2.2E−04	6.4E−04	4.2E−02
Ni	–	–	2.1E−02	7.2E−02	4.5E−02	1.5E−01	5.5E−02	1.9E−01
Pb	3.6E−03	–	–	3.9E−02	3.1E−03	1.6E−03	1.5E−03	6.6E−02
Sr	1.6E−03	2.3E−04	5.3E−04	1.7E+00	3.5E−02	8.8E−02	3.9E−02	2.1E−01
U	1.0E−03	9.5E−06	7.7E−05	1.1E−04	6.1E−04	3.4E−04	1.3E−03	1.5E−02
Zr	1.7E−04	–	1.7E−04	1.2E−05	1.9E−04	1.7E−04	1.8E−04	5.3E−04

B								
Element	Freshwater fish from the site			Reference pelagic fish	Sea mollusc from the site			Reference mollusc
	Pike	Roach	Tench		Baltic macoma	Lagoon cockle	River nerite	
Cl	3.2E+01	1.4E+02	5.1E+01	8.2E+01	2.3E−01	–	3.5E−01	4.6E−02
Cs	5.3E+03	1.5E+03	1.8E+03	7.1E+03	4.9E+02	2.1E+02	3.4E+02	6.6E+01
I	5.0E+01	–	–	1.8E+02	5.4E+01	–	2.7E+02	1.4E+01
Nb	9.7E+00	–	1.2E+01	2.3E+02	1.7E+02	1.0E+03	3.6E+02	6.4E+03
Se	5.3E+02	2.1E+02	8.4E+02	2.0E+02	1.4E+04	3.0E+03	6.0E+03	1.7E+03
Sr	5.0E+00	1.3E+01	4.2E+00	1.7E+01	1.2E+03	7.0E+02	1.8E+03	5.0E+03
U	6.6E−02	4.3E−01	2.2E−01	3.0E+01	2.1E+04	8.2E+03	3.4E+04	5.1E+02
Zr	–	1.3E+02	–	3.0E+02	1.7E+04	9.8E+03	1.4E+04	4.6E+03

^a^Small or large mammal

## Discussion

The dose rates for all investigated ERICA reference organisms were found to be several orders of magnitude below the screening dose rate of 10 μGy h^−1^. This suggests that the release under the central corrosion case would be of negligible concern for the protection of non-human biota in the Forsmark area. The uncertainty of calculated dose rates did not affect these results significantly.

It should be noted that the future potential release of radioactivity from the planned repository, and the resulting activity concentrations in the environment, is the main driver behind the calculated dose rates in this assessment, and consequently the dose rates are expected to scale more or less linearly to the potential release term. In the SKB (2011) safety assessment, the concentrations in the environmental media were calculated to several potential discharge areas during an interglacial episode. Uncertainties with respect to the location and timing of the release were treated cautiously in these simulations (Avila et al. 2013). However, the overall uncertainty in model endpoints was dominated by parameter uncertainty, and a combined evaluation of system and model uncertainties indicated that the model results (including the concentration in environmental media) were not overly conservative (Avila et al. 2010).

A comparison of transfer parameters and the limited effect of size and morphology on absorbed doses (Torudd 2010) suggest, as in earlier studies (Vives i Batlle et al. 2011), that the reference organisms provide a sound representation of the species of interest at the site. However, the evaluation of transfer parameters highlighted the importance of collecting sufficient measurements from the site. That is, the representation of a number of radionuclides and organism groups were limited in the generic data, and the reported variation in CR values for reference organisms did not always capture observations for individual species and radionuclides observed at the site. Moreover, there were a few cases where the CR of well-represented organisms (e.g., vascular plants, mammals, and fish) and radionuclides (e.g., Cs, Pb, and U) differed systematically between site data and the ERICA database by an order of magnitude (Table 3), suggesting site-specific relationships between environmental and organism concentrations (see Torudd 2010 for a detailed discussion). Nevertheless, neither a re-analysis of the reference organisms using CR values from the site (as far as possible) nor an analysis based on species morphologies and CR values from organisms observed at the site (as far as possible) affected the results in any significant way (Torudd 2010). That is, these dose rates, too, were at least 4 orders of magnitude below the “screening” dose rate.

The SKB safety assessment (SKB 2011) found that the most significant release of radionuclides to the biosphere would result from the corrosion scenario, i.e., canister failure due to corrosion. The consequences for biota were calculated for the central calculation cases of the corrosion scenario, but the conclusions can be generalized to encompass all variants of the corrosion scenario. This is because the predicted release rates of dose-contributing nuclides in the different release scenarios (and calculation cases) vary by less than an order of magnitude (see SKB 2011, Sects. 13.5.7 and 13.6.5) and calculated dose rates were several orders of magnitude below the screening dose rate.

In a similar study relating to a repository for spent nuclear fuel which is planned at Olkiluoto, Finland, Smith and Robinson (2006) identified some data gaps, but concluded that the dose rates predicted for all organism types were several orders of magnitude below those at which population effects would be expected and, accordingly, below those at which effects on the individual may be anticipated. These general results agree with the results obtained in this study.

Similarly, Robinson et al. (2010) concluded that post-closure releases of radionuclides from a generic geological disposal facility are unlikely to give rise to discernible effects to individual organisms, populations, or communities. They do, however, point out that the methodology still has some limitations. For instance, the reference organism approach does not as yet allow for indirect effects resulting from interactions (e.g., between different species). They consider that inherent uncertainties of the input data, e.g., CRs, also merit further study.

A more complete and final study of the Olkiluoto case by Hjerpe and Broed (2010) was performed using the full ERICA approach and tool, i.e., in a manner directly comparable to this study. Their results concerning dose rates were similar and they concluded that any radiological consequences of releases from the repository would be negligible. Hjerpe and Broed (2010) listed several remaining issues that were expected to require further work. Issues that are relevant for the safety assessment of the planned Forsmark repository include the use of ecosystem models versus a transfer factor approach in radionuclide transport modeling, management of uncertainties, and difficulties in applying geometrical constraints, such as the ellipsoidal geometry (especially for plants) in the assessment of dose to non-human biota.

The last two issues, uncertainty management and geometrical constraints, are applicable also to the present assessment of non-human biota. For example, in this assessment, the maximum environmental concentrations across multiple discharge areas and points in time were cautiously used, instead of explicitly managing the uncertainties in the calculated environmental activity concentrations. Difficulties in the translation of species morphology to ellipsoidal geometries were also encountered for a few organism groups. However, morphology was shown to have little or insignificant effect on calculated dose rates of the organisms used in the assessment (Torudd 2010).

## Conclusion

Given that dose rates for all investigated organisms are far below the screening dose rate of 10 μGy h^−1^, and the fact that identified uncertainties were found to have no significant effect on these results, it follows that a potential release from the repository is highly unlikely to cause detrimental effects on the survival and reproduction of individual organisms. This conclusion can be generalized to the two significant release scenarios, and encompasses endangered species, species that are of economic or biological importance, as well as species that are important for ecosystem function. As no effects are expected at the level of the individual organism, effects at the levels of populations, communities, and ecosystems are also highly unlikely.

Thus, from this assessment it is concluded that neither negative effects of the repository on biodiversity nor sustainable use of natural resources in the Forsmark area are of concern. Nevertheless, technical refinements of the methods used to assess the safety of non-human biota are ongoing, and international developments should be kept under review during the repository construction phase and methods of analysis refined if and as required.

## Electronic Supplementary Material

Below is the link to the electronic supplementary material.

Supplementary material 1 (PDF 73 kb)
