# Supplementary material for: Assessment of Long-Term Radiological Effects on Plants and Animals from a Deep Geological Repository: No Discernible Impact Detected
Source: Ambio. 2013 Apr 26;42(4):506–16. doi: 10.1007/s13280-013-0403-9 (PMC3636368; doi:10.1007/s13280-013-0403-9)
Supplement: Supplementary file 1 — Supplementary material 1 (PDF 73 kb) [file 13280_2013_403_MOESM1_ESM.pdf]

Electronic Supplementary Material

Assessment of Long-Term Radiological Effects on Plants and Animals from a Deep Geological Repository: No Discernible Impact Detected

Jesper Torudd and Peter Saetre

APPENDIX

Activity concentrations in terrestrial, freshwater, and marine/brackish ecosystems, resulting from a potential release of radionuclides from a planned nuclear waste repository in Forsmark, are provided in Table S1.

The table shows maximum values across 17 potential discharge areas for radionuclide-bearing groundwater, developing over an interglacial episode.

For each radionuclide and ecosystem, maximum concentrations are listed for environmental media corresponding to ERICA habitats, with an approximate time.

All radionuclides are included in the database of the ERICA Tool, but those shown in *italics* were not part of the default set at the time of the assessment.

Table S1 Activity concentrations in terrestrial, freshwater, and marine/brackish ecosystems after a potential release of radionuclides (modified from Torudd 2010):

	Terrestrial			Fresh water			Sea		
Nuclides	Soil (Bq/kg dw)	Air (Bq/m^3)	Time (AD)	Sediment (Bq/kg dw)	Water (Bq/l)	Time[a] (AD)	Sediment (Bq/kg dw)	Water (Bq/l)	Time[a] (AD)
**Am-241**	2.2E-12	1.1E-19	1600	3.7E-11	1.1E-16	3100	1.3E-12	8.4E-19	1500
Am-243	7.6E-10	3.8E-17	2700	1.9E-08	7.7E-14	3100	2.5E-10	2.6E-16	2500
**C-14**	4.7E-10	1.4E-13	2700	9.5E-09	2.8E-11	3100	4.1E-09	6.4E-12	2500
**Cl-36**	1.9E-06	9.5E-14	2700	1.5E-06	2.0E-08	3100	2.3E-06	1.1E-09	2500
Cm-245	7.1E-11	3.5E-18	7500	9.7E-10	1.7E-15	3100	1.5E-11	4.3E-18	7500
**Cs-135**	8.2E-04	4.1E-11	7500	1.8E-03	7.5E-09	3100	2.1E-05	7.3E-11	7500
**I-129**	8.7E-04	4.4E-11	2700	3.8E-02	4.9E-07	3200	9.3E-04	1.1E-08	2500
**Nb-94**	8.4E-03	4.2E-10	7500	2.6E-02	2.7E-08	3100	4.1E-04	5.0E-10	7500
**Ni-59**	8.7E-01	4.3E-08	2700	1.2E+01	8.1E-05	3100	1.6E-01	7.4E-07	2500
**Np-237**	6.2E-03	3.1E-10	2700	2.5E-01	2.9E-06	3200	5.2E-03	6.7E-08	2500
**Pb-210**	2.9E-03	1.4E-10	1100	8.5E-02	1.6E-08	2100	1.2E-03	4.3E-10	1000
**Po-210**	2.9E-03	1.4E-10	1100	8.6E-02	1.6E-07	3100	1.3E-03	5.2E-11	1000
**Pu-239**	1.6E-04	7.9E-12	2700	2.1E-03	5.3E-08	3100	4.3E-05	1.3E-10	2500
**Pu-240**	2.7E-09	1.4E-16	2700	6.7E-08	1.1E-12	3100	1.4E-09	4.1E-15	2500
Pu-242	5.9E-04	2.9E-11	2700	4.5E-03	2.0E-07	3100	9.3E-05	2.8E-10	2500
**Ra-226**	2.8E-03	1.4E-10	2700	7.5E-02	2.7E-07	3200	1.2E-03	7.2E-09	2500
**Se-79**	1.2E-04	5.9E-12	2700	3.0E-03	6.1E-08	3100	9.7E-05	1.6E-09	2500
**Tc-99**	1.9E-04	9.7E-12	2700	1.5E-03	3.6E-06	3100	6.7E-04	1.5E-07	2500
Th-229	1.0E-03	5.0E-11	7500	5.1E-03	5.7E-09	3100	1.7E-04	9.5E-12	7500
**Th-230**	2.4E-06	1.2E-13	2700	2.9E-06	1.1E-11	3100	6.8E-08	4.2E-15	2500
**Th-232**	1.8E-11	9.1E-19	7500	3.1E-11	1.1E-16	2600	3.8E-13	2.3E-20	7500
U-233	1.4E-03	6.8E-11	7500	1.5E-02	6.5E-08	3100	1.7E-04	1.1E-09	7500
**U-234**	8.6E-06	4.3E-13	7500	4.6E-05	4.0E-10	3100	5.1E-07	3.0E-12	7500
**U-235**	5.7E-07	2.9E-14	7500	2.9E-06	2.7E-11	3100	3.2E-08	1.9E-13	7500
U-236	9.4E-06	4.7E-13	2700	4.9E-05	4.4E-10	3100	5.5E-07	3.2E-12	2500
**U-238**	6.1E-06	3.1E-13	7500	3.2E-05	2.9E-10	3100	3.6E-07	2.1E-12	7500
Zr-93	1.5E-01	7.4E-09	7500	1.2E+00	7.5E-06	3100	2.7E-02	1.1E-08	7500

a = time for maximum concentration in water

BOX S1: DOSE ASSESSMENTS USING THE ERICA TOOL

The ERICA Tool is a software package that supports the ERICA Integrated Approach to assessing the safety of the environment (Brown et al. 2008). The approach provides guidelines on problem formulation, impact assessment, and data evaluation. The ERICA Tool guides the user through the assessment process, keeps records, and performs the calculations to estimate whole body dose rates to selected organisms.

Dosimetry

Radionuclides in the environment lead to both internal and external exposure of organisms. In the ERICA Tool, the *internal* absorbed dose rate (μGy h^{-1}) in biota is a function of whole-body activity concentration, size of the organism and the types, yields, and energies of emitted radiations. Absorbed dose rate from *external* radiation depends not only on organism size and the types, yields, and energies of emissions, but also on the contamination level in and the properties of the environment, but is not dependent on the activity concentration in the organism.

The ERICA Tool considers the dosimetry of the majority of the radionuclides included in ICRP Publication 38 (ICRP, 1983), and interacts with the FREDERICA database on radiation effects (Copplestone et al. 2008), which is a compilation of the scientific literature on radiation effect experiments and field studies. Below is a brief description of the methods used for calculating dose conversion factors in the ERICA Tool. A detailed description of the underlying approaches and the data that have been applied in the dosimetric module of the ERICA Tool is presented in Ulanovsky et al. (2008).

Calculation of dose conversion coefficients

Dose coefficients are quantities linking amounts or concentrations of activity to doses or dose rates. In the ERICA Tool, two sets of Dose Conversion Coefficients (DCCs) are defined: for doses due to intakes, DCC_{int} is defined as the internal absorbed dose rate (μGy h^{-1}) per unit activity concentration in an organism (Bq kg^{-1} fw) and for doses due to exposures from surrounding media, DCC_{ext} is defined as the external absorbed dose rate (μGy h^{-1}) per unit concentration in environmental media (Bq kg^{-1} or Bq l^{-1} fw) (Pröhl 2003, Brown et al. 2008). Using DCC_{int} and DCC_{ext}, internal and external dose rates to an organism can be computed; the total dose rate to an organism is obtained as the sum of these dose rates.

Assessment of results

The assessment element is organized in three separate *tiers*. When the effects are not shown to be negligible in a lower tier, the assessment should continue to the next tier.

Tier 1 assessment

This is a simple and ‘conservative’ assessment to exclude situations of negligible concern from further evaluation. Tier 1 does not permit the addition of any other radionuclides or species than the default sets provided by the ERICA Tool, and therefore was not used in the present analysis.

Tier 2 assessment

To permit the use of site-specific organism data (CR values and geometries) and the addition of some radionuclides, Tier 2 was used as the entry point in the present assessment. In Tier 2, Risk Quotients (RQ) are calculated for each organism and each radionuclide according to the relationship:

$$RQ_i = D_i / D_{lim}$$

where D_i = the estimated whole-body absorbed dose rate to organism *i*, and D_{lim} = the projected no-effect (‘screening’) dose rate. The outputs from the Tier 2 assessments are 1) ‘expected’ RQs obtained by deterministic calculations using mean values for all parameters, and 2) ‘conservative’ RQs (95th percentile, assuming an exponential distribution of dose rates). If some ‘conservative’ RQ’s exceed one, the results and assessments need to be reviewed, and if any ‘expected’ RQ exceeds one, then further assessment using Tier 3 is warranted.

Tier 3 assessment

Tier 3 is a probabilistic risk assessment in which uncertainties within the results may be determined using sensitivity analysis. The assessor can also access up-to-date scientific literature on the biological effects of exposure to ionizing radiation in a number of different species. Together, these allow the user to estimate the probability (or incidence) and magnitude (or severity) of the environmental effects

likely to occur and, by discussion and agreement with stakeholders, to determine the acceptability of the risk to non-human species.

Situations that require a Tier 3 assessment are likely to be complex and unique. The assessment must be adapted to the particular conditions, and will not provide a simple yes/no answer.

REFERENCES

Brown, J.E., B. Alfonso, R. Avila, N.A. Beresford, D. Copplestone, G. Pröhl, and A. Ulanovsky. 2008. The ERICA Tool. *Journal of Environmental Radioactivity* 99: 1371–1383.

Copplestone, D., J. Hingston, and A. Real. 2008. The development and purpose of the FREDERICA radiation effects database. Journal of Environmental Radioactivity 99: 1456-1463.

ICRP. 1983. Radionuclide transformations: Energy and intensity of emissions ICRP Publication 38. Annals of the ICRP 11–13.

Pröhl G. (ed.). 2003. Dosimetric models and data for assessing radiation exposure to biota. Deliverable 3 Report of the FASSET project. European Commission. 103 pp. Retrieved January 4, 2012, from https://wiki.ceh.ac.uk/display/rpemain/FASSET+reports

Torudd, J. 2010. Long term radiological effects on plants and animals of a deep geological repository. SR-Site Biosphere. Svensk Kärnbränslehantering AB, SKB TR-10-08, Stockholm, Sweden, Report, 84 pp.

Ulanovsky. A., G. Pröhl, and J.M. Gómez-Ros. 2008. Methods for calculating dose conversion coefficients for terrestrial and aquatic biota. Journal of Environmental Radioactivity 99: 1440–1448.
